# Complete Recovery of an Occluded Coronary Artery Secondary to Spontaneous Coronary Artery Dissection With Medical Management in a Young Patient Presenting With Acute Coronary Syndrome

**DOI:** 10.7759/cureus.29980

**Published:** 2022-10-06

**Authors:** Hafiz Khan, Amman Yousaf, Muhammad Ahmad, Ahmad Munir, Ankush Moza

**Affiliations:** 1 Cardiovascular Department, McLaren Flint/Michigan State University, Flint, USA; 2 Internal Medicine Department, McLaren Flint/Michigan State University, Flint, USA

**Keywords:** acute coronary syndrome, atherosclerosis, coronary artery angiography, spontaneous coronary dissection, medical management

## Abstract

Spontaneous coronary artery dissection* *(SCAD) is defined as a tear in the coronary arterial wall. The clinical presentation is similar to acute coronary syndrome (ACS); however, most of the patients are usually younger and do not have typical risk factors such as atherosclerosis. In addition, the management of SCAD varies from case to case unlike that of ACS due to atherosclerotic plaque rupture; therefore, recognizing and treating it appropriately is crucial. We present a case of a 47-year-old female who presented with typical clinical findings of ACS and was diagnosed with occlusion of the left anterior descending coronary artery due to SCAD on emergent coronary angiography. The patient was treated with medical management only, and a repeat coronary angiography showed complete healing of the vessel wall after six weeks. This article highlights that early diagnosis, recognition, and medical management of SCAD can prevent unnecessary invasive intervention.

## Introduction

Spontaneous coronary artery dissection (SCAD) is an acute non-iatrogenic tear in the coronary arterial wall, which leads to disruption of coronary blood flow and subsequent myocardial infarction [1]. SCAD, however, can also result from an intramural hemorrhage causing separation of the layers of an epicardial coronary artery. It is not associated with atherosclerosis or trauma; it is the formation of an intramural hematoma resulting from SCAD that leads to myocardial injury [1,2]. SCAD likely accounts for up to 1% to 4% of overall acute coronary syndrome (ACS) cases [1]. We present a 46-year-old female who presented to the hospital with typical cardiac chest pain and new-onset ST elevations and was diagnosed with SCAD on percutaneous coronary intervention. This article emphasizes the medical management of SCAD with favorable outcomes.

## Case presentation

A 47-year-old female patient with no significant past medical history presented with sudden onset substernal chest pain after significant emotional trauma to the emergency department. The pain started at rest about an hour before the presentation, progressively increasing in intensity, 9/10 in intensity, non-radiating, and the patient described it as crushing in nature. Her presenting vitals were heart rate of 97 beats per minute, blood pressure of 123/78 mmHg, respiratory rate of 19 breaths per minute, and temperature of 98.8 Fahrenheit. Basic labs were withdrawn, including complete blood count, renal function tests, liver function tests, and serum electrolytes.

**Figure 1:**
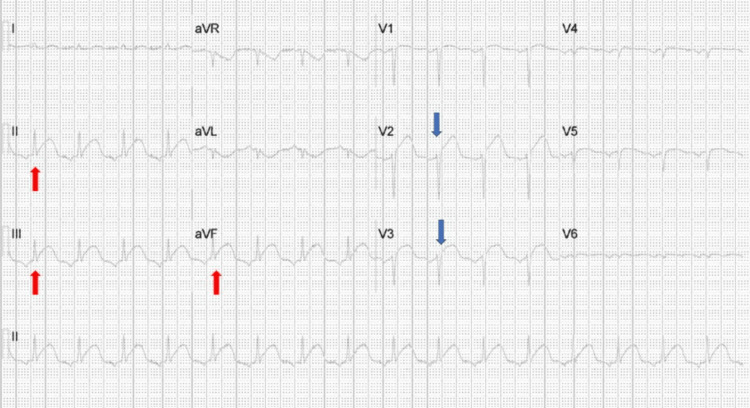
Twelve-lead electrocardiogram showing ST elevations in anterior (blue arrows) and inferior (red arrows) leads

A 12-lead EKG was performed, which showed anterior and inferior leads ST elevations that warranted emergency percutaneous coronary angiography (Figure 1).

**Figure 2:**
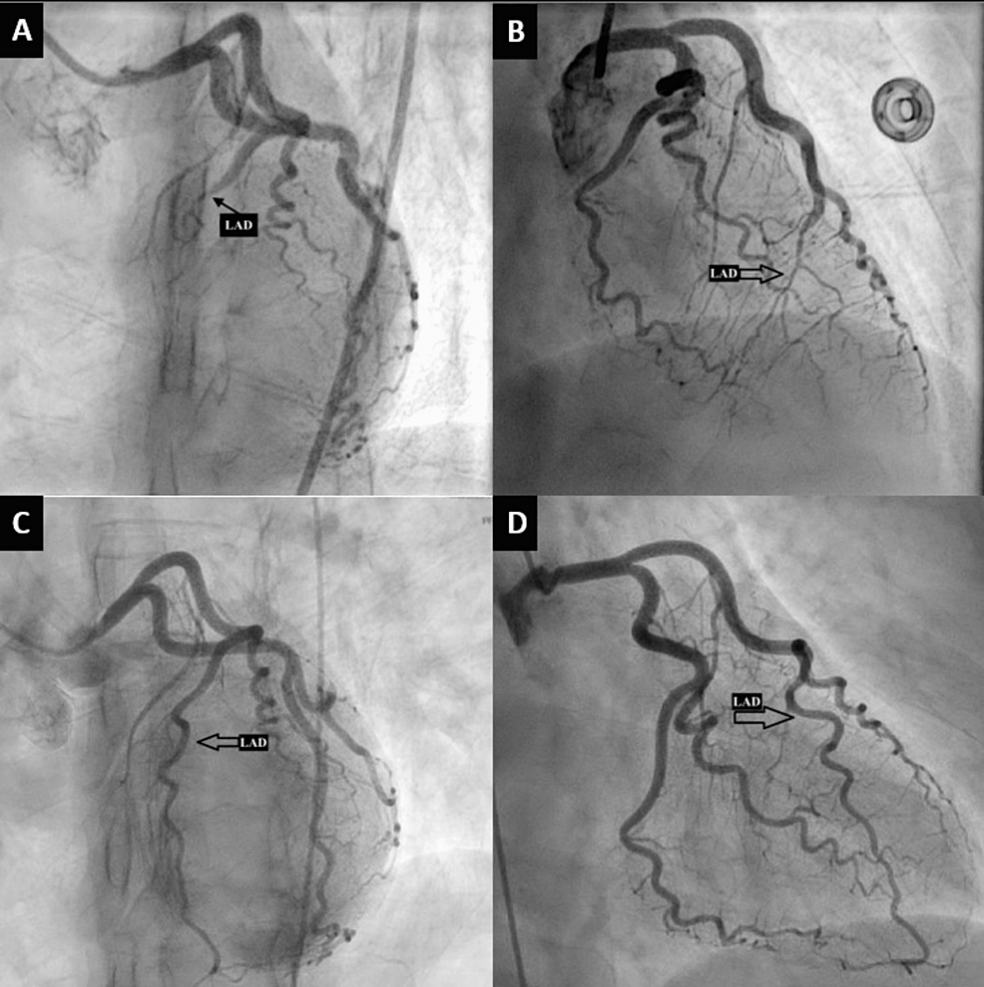
Cardiac catheterization images (A) LAO caudal view & (B) RAO cranial view showing mid-LAD occlusion. (C) LAO caudal & (D) RAO cranial view showing open LAD. (Abbreviations: LAO; left anterior oblique, RAO; right anterior oblique, LAD; left anterior descending)

Coronary angiography showed subtotal occlusion of the mid-left anterior descending artery with angiographic findings suggestive of spontaneous coronary artery dissection (Figure 2).

Despite subtotal occlusion and wall motion abnormalities in the distal anterior and apical segments on the left ventriculography, a decision was made not to proceed with coronary intervention, as the patient was hemodynamically and electrically stable with overall normal LV function. The patient was medically treated with aspirin, clopidogrel, atorvastatin, therapeutic intravenous heparin, beta-blockers, and angiotensin-converting enzyme (ACE) inhibitors. Initial cardiac enzymes (troponin T) on presentation were 0.22 ng/dL (normal 0.00-0.04 ng/dL), which increased to as high as 43.11 ng/dL (normal 0.00-0.04 ng/dL) by the next day. The rest of the withdrawn labs were within normal limits. A transthoracic echocardiogram confirmed the findings with LVEF of 65% (modified Simpsons method), distal anterior, and apical akinesis with hyperdynamic LV systolic function of the rest of the ventricle.

The patient was discharged on day 3 after the troponins trended down to 0.01 ng/dL on outpatient follow-up. The patient was asymptomatic in a two-week follow-up period. After six6 weeks, the patient had a repeat echocardiogram, which revealed normal wall motion of the LV after which cardiac catheterization was performed, which showed complete healing of the vessel lumen likely due to resolution of intramural hematoma, one of the mechanisms of SCAD. The patient has had no recurrence of chest pain to date.

## Discussion

The first case of SCAD was illustrated by Petty et al. in a 42-year-old woman [3]. The presentation of SCAD can be similar to that of ACS. As more and more data has emerged, the recommendations for managing acute MI caused by SCAD now differ vastly from that for atherosclerotic myocardial infarction [2,4]. It is predominant among younger females who usually lack atherosclerosis. Other documented risk factors in the literature are fibromuscular dysplasia and emotional or physical stress [1-2,4]. There are two main theories regarding the pathogenesis of SCAD. One theory proposes that an inciting event causes an intimal tear, which allows blood to leak into the vessel wall from the lumen. Another theory proposes that spontaneous hemorrhage from the vessel itself could lead to separation of the vessel walls and subsequent obstruction to blood flow. In the second case, no tear in the intima of the vessel is identified [2,5].

In one retrospective study by Sharma et al., there was female predominance (87%); 90% of patients presented with chest pain or discomfort, 57% with NSTEMI, and 43% with STEMI [6]. The diagnosis of SCAD requires a high index of suspicion, especially in a young woman without any traditional risk factors for atherosclerosis [1]. Coronary angiography is a gold standard for confirming the diagnosis and presence of SCAD. If coronary angiography is inconclusive, other imaging modalities, such as intravascular ultrasonography, optical coherence tomography (OCT), and coronary computed tomography angiography (CCTA) should be pursued. Diagnosing SCAD early in the course is crucial, as treatment and long-term management differ from MI [4]. Angiographically, SCAD has three distinct patterns (Table 1).

**Table 1:** Types of Spontaneous Coronary Artery Dissection

Types	Features
Type I	Contrast staining arterial wall and multiple radiolucent lumens [7].
Type II	Most common. Diffuse stenosis is usually greater than 20 mm in length. Commonly involves the mid to distal segments of the coronary arteries [7].
Type III	Commonly involves focal or tubular stenosis, usually less than 20 mm in length. Most likely to be misdiagnosed [7].

The underlying pathophysiology of SCAD is medial dissection. Luminal obstruction from SCAD is due to compression from a hematoma within the vessel media, and often, lesions can be diffuse and long. This leads to an unpredictable response to vasodilation from PCI. Tweet et al. illustrated that with conservative therapy, revascularization had a markedly elevated risk of requiring emergency CABG because of PCI failure. Most patients treated in the conservative group had an uneventful hospital course [7,8]. The role of antiplatelet and beta-blockers therapy in SCAD is not well-studied and is generally continued. However, statins and anticoagulation are generally discouraged due to the lack of any clear benefits [1-3,9]. Unstable patients having ventricular arrhythmias, recurrent chest pain, cardiogenic shock, extensive dissection involving proximal vessels, or dissection of the left main should be considered for revascularization [7,9].

## Conclusions

In conclusion, spontaneous coronary artery dissection is a non-atherosclerotic occlusion of the coronary arteries that usually presents as acute coronary syndrome in a relatively younger population without underlying risk factors for coronary artery disease. Cardiovascular interventions are not usually needed and medical management has been recommended with favorable outcomes. This article also emphasizes the need for further research regarding medical management vs. emergent intervention for this rare cardiac emergency.
